# COVID-19 Associated Pulmonary Aspergillosis (CAPA)—From Immunology to Treatment

**DOI:** 10.3390/jof6020091

**Published:** 2020-06-24

**Authors:** Amir Arastehfar, Agostinho Carvalho, Frank L. van de Veerdonk, Jeffrey D. Jenks, Philipp Koehler, Robert Krause, Oliver A. Cornely, David S. Perlin, Cornelia Lass-Flörl, Martin Hoenigl

**Affiliations:** 1 Center for Discovery and Innovation, Hackensack Meridian Health, Nutley, NJ 07110, USA; david.perlin@hmh-cdi.org; 2 Life and Health Sciences Research Institute (ICVS), School of Medicine, University of Minho, 4710-057 Braga, Portugal; 3 ICVS/3B’s—PT Government Associate Laboratory, 4710-057 Braga, Portugal; 4 Department of Internal Medicine, Radboud University Medical Center, 6525 Nijmegen, The Netherlands; frank.vandeveerdonk@radboudumc.nl; 5 Radboud Institute of Molecular Life Sciences, Radboud University Medical Center, 6525Nijmegen, The Netherlands; 6 Department of Medicine, University of California San Diego, San Diego, CA 92103, USA; jjenks@ucsd.edu; 7 Clinical and Translational Fungal-Working Group, University of California San Diego, La Jolla, CA 92093, USA; 8 Department I of Internal Medicine, Medical Faculty and University Hospital Cologne, University of Cologne, 50937 Cologne, Germany; philipp.koehler@uk-koeln.de (P.K.); oliver.cornely@uk-koeln.de (O.A.C.); 9 Cologne Excellence Cluster on Cellular Stress Responses in Aging-Associated Diseases (CECAD), University of Cologne, 50937Cologne, Germany; 10 Section of Infectious Diseases and Tropical Medicine, Department of Internal Medicine, Medical University of Graz, 8036 Graz, Austria; Robert.krause@medunigraz.at; 11 Zentrum fuer klinische Studien (ZKS) Köln, Clinical Trials Centre Cologne, 50937 Cologne, Germany; 12 German Center for Infection Research (DZIF), Partner Site Bonn-Cologne, Medical Faculty and University Hospital Cologne, University of Cologne, 50937 Cologne, Germany; 13 Division of Hygiene and Medical Microbiology, Medical University of Innsbruck, 6020 Innsbruck, Austria; cornelia.lass-floerl@i-med.ac.at; 14 Division of Infectious Diseases and Global Public Health, Department of Medicine, University of California, San Diego, San Diego, CA 92093, USA

**Keywords:** SARS COV-2, *Aspergillus*, novel coronavirus, superinfection, co-infection, risk factors, prevalence, challenges, immune response, expert statement, European Confederation of Medical Mycology

## Abstract

Like severe influenza, coronavirus disease-19 (COVID-19) resulting in acute respiratory distress syndrome (ARDS) has emerged as an important disease that predisposes patients to secondary pulmonary aspergillosis, with 35 cases of COVID-19 associated pulmonary aspergillosis (CAPA) published until June 2020. The release of danger-associated molecular patterns during severe COVID-19 results in both pulmonary epithelial damage and inflammatory disease, which are predisposing risk factors for pulmonary aspergillosis. Moreover, collateral effects of host recognition pathways required for the activation of antiviral immunity may, paradoxically, contribute to a highly permissive inflammatory environment that favors fungal pathogenesis. Diagnosis of CAPA remains challenging, mainly because bronchoalveolar lavage fluid galactomannan testing and culture, which represent the most sensitive diagnostic tests for aspergillosis in the ICU, are hindered by the fact that bronchoscopies are rarely performed in COVID-19 patients due to the risk of disease transmission. Similarly, autopsies are rarely performed, which may result in an underestimation of the prevalence of CAPA. Finally, the treatment of CAPA is complicated by drug–drug interactions associated with broad spectrum azoles, renal tropism and damage caused by SARS-CoV-2, which may challenge the use of liposomal amphotericin B, as well as the emergence of azole-resistance. This clinical reality creates an urgency for new antifungal drugs currently in advanced clinical development with more promising pharmacokinetic and pharmacodynamic profiles.

## 1. Introduction

Invasive fungal infections caused by various fungal genera, including *Aspergillus*, complicate and endanger lives of millions of individuals annually [1]. *Aspergillus* genera, most frequently *Aspergillus fumigatus*, are ubiquitous in the environment and cause a wide range of infections in humans, including invasive pulmonary aspergillosis (IPA), chronic pulmonary aspergillosis (CPA), allergic bronchopulmonary aspergillosis (ABPA), chronic rhinosinusitis, fungal asthma, and *Aspergillus* bronchitis [2,3]. IPA, the most severe manifestation of disease from *Aspergillus*, is associated with high mortality rates and is a prominent complication among those with profound immunosuppression, such as those undergoing hematopoietic transplantation, as well as those with structural lung damage who receive systemic corticosteroids for their underlying condition, such as patients with chronic obstructive pulmonary diseases (COPD) [2].

Recently, it has been reported that a relatively high number of influenza patients presenting with severe acute respiratory distress syndrome (ARDS) also rapidly develop IPA, which is associated with increased duration of hospitalization and mortality [4,5]. Corticosteroid use and pulmonary epithelial damages caused by severe influenza are the main risk factors for developing IPA [4,5]. The recent global pandemic of coronavirus disease-19, also known as COVID-19, has infected over 6 million patients worldwide, with more than 360,000 deaths. It has been shown that up to 40% of COVID-19 hospitalized patients can develop ARDS [6], and thereby become susceptible to acquire co-infections caused by bacteria and also *Aspergillus* spp. [7,8], although frequency of co-infections seems to vary between centers and overall co-infections may occur less frequently than with severe influenza [9]. Once they occur, these superinfections are associated with high mortality rates and may prolong the acute phase of COVID-19 [10]. In this comprehensive review, we discuss various aspects of COVID-19 associated pulmonary aspergillosis (CAPA), focusing specifically on immunology, risk factors, prevalence, diagnosis, treatment, and current challenges.

## 2. Immunology

Dissecting the complex pathogenesis of CAPA requires a molecular understanding of the physiological processes whereby infection with SARS-CoV-2 facilitates fungal pathogenesis. Similar to other SARS coronaviruses, SARS-CoV-2 targets and invades epithelial cells and type II pneumocytes through binding of the SARS spike protein to the angiotensin-converting enzyme 2 (ACE2) receptors [11]. Cleavage of the S1/S2 domain by the type 2 transmembrane protease TMPRSS2 leads to the activation of the spike protein [12], thereby facilitating viral entry into the target cell via ACE2. Besides its role as a SARS virus receptor, ACE2 was also demonstrated to be required for protection from severe acute lung injury in ARDS [13]. In support of this, an insertion/deletion polymorphism that affects ACE activity was associated with ARDS susceptibility and outcome [14]. Whether the preceding interaction of SARS-CoV-2 with host cells, by disrupting the regulation of the renin-angiotensin system and or the kallikrein-kinin system, contributes to the development of CAPA, is not known.

Viral entry and infection elicit an immune response, which is initiated by the establishment of an inflammatory cascade by innate immune cells. Although the receptor(s) and signaling pathways involved in the immune recognition of *Aspergillus* and the downstream production of inflammatory mediators are relatively well characterized [15], not much is known regarding how the immune system senses and responds to SARS-CoV-2. Based on the available knowledge for infections with other coronaviruses, two possible mechanisms can be anticipated and are likely to explain the development of ARDS and consequently CAPA. The first involves the release of danger-associated molecular patterns (DAMPs), signal molecules released by dying or damaged cells that act as endogenous danger signals to promote and exacerbate the immune and inflammatory response leading to lung injury [16]. It is noteworthy that DAMPs have also been shown to regulate inflammation in fungal diseases [17]. The DAMP/receptor for advanced glycation end-products axis was found to integrate with Toll-like receptors (TLRs) to generate and amplify the inflammatory response in experimental aspergillosis [18]. Moreover, recipients of allogeneic stem-cell transplantation harboring genetic variants underlying a hyperactivation of danger signaling in response to infection displayed an increased risk of developing IPA [19]. This emerging concept could help explain fungal pathogenesis in conditions of exuberant inflammation such as that observed in COVID-19 patients and highlights DAMP targeting as potential immunomodulatory strategy in CAPA.

A second possibility involves the collateral effects of recognition pathways required for the activation of antiviral immunity that may, paradoxically, contribute to an inflammatory environment that favors secondary infections. ACE2 is not well expressed on immune cells and SARS-CoV are recognized by TLR4 and TLR3, leading to the activation of MyD88- or TRIF-mediated signaling, respectively [20,21]. Of note, this may be potentiated in the presence of *Aspergillus spp.* which activate TLR4/MyD88/TRIF through the cleavage of fibrinogen [22]. It is likely that SARS-CoV-2 may elicit, to a large extent, overlapping signaling pathways towards the production of inflammatory cytokines. In addition, the activation of the inflammasome by SARS-CoV and the consequent production of IL-1β is an event that contributes further to the hyperinflammatory response [23]. A transcriptome analysis of COVID-19 patients revealed an early immune response characterized by a marked upregulation of the IL-1 pathway, even after respiratory function nadir [24]. The possibility that IL-1 and related pro-inflammatory pathways could serve as therapeutic targets was demonstrated by the favorable responses in severe COVID-19 patients with secondary hemophagocytic lymphohistiocytosis treated with the interleukin-1 receptor antagonist anakinra [25]. Similar findings were also disclosed in acute leukemia patients with COVID-19 [26]. Likewise, IL-1 blockade with anakinra has also been found to ameliorate inflammation in both chronic granulomatous disease [27] and cystic fibrosis [28], and in either case, to restrain susceptibility to infection or colonization by *Aspergillus*. Therefore, the early hyperactivation of the IL-1 pathway induced by the SARS-CoV-2 infection may be a major factor establishing a highly permissive inflammatory environment that favors fungal pathogenesis.

Besides IL-1, increased levels of IL-6 have also been consistently reported in severe cases of COVID-19 [29,30], with an impact on immune cell function and the anti-viral mechanisms of immune cells [31]. An enhanced production of IL-6 is also observed in epithelial cells following infection with *A. fumigatus*, suggesting that, at least in some patients, the co-infection may contribute to the increased levels of this cytokine in severe COVID-19 patients [32]. In a large patient series of COVID-19 patients with ARDS, the use of the IL-6 receptor antagonist tocilizumab was recently reported to promote rapid and sustained responses associated with significant clinical improvement [33]. However, such clinical approach could paradoxically enhance the predisposition to CAPA, similar to animal models of IL-6 deficiency subjected to experimental aspergillosis [34]. For this reason, ongoing trials are addressing the combined use of IL-6 antagonists and antifungal prophylaxis in severe COVID-19 patients.

An emerging body of evidence supports therefore an increased systemic inflammatory reaction in patients with severe SARS-CoV-2 infection who are more likely to develop CAPA. In this regard, increased levels of circulating proinflammatory cytokines, such as TNF, were observed in patients requiring intensive care, compared to those with milder infections [35]. Other studies, however, have also unveiled marked defects in immune cell populations, namely T-lymphocytes, as another factor explaining the immune dysfunction in patients with COVID-19 [36]. This suggests that while sustained innate immune function leads to hyperinflammation [37], lymphocyte numbers decline, and their function may be defective. In this regard, severe lymphocytopenia was among the factors in a risk score model that predicted the development of invasive mold disease in patients with hematological malignances [38]. It is thus reasonable to speculate that in elderly individuals or with co-morbidities, defective immune responses to SARS-CoV-2 may allow unrestricted viral replication which, in turn, elicits hyperinflammation and severe complications such as ARDS [39], besides establishing favorable conditions for the acquisition of secondary infections, such as CAPA.

While there is much to be learned about CAPA, our current understanding of the pathophysiology of other coinfections with respiratory viruses such as influenza [40] provides an important framework towards the effective design of immunotherapeutic approaches and the identification of the patients that could benefit the most from them.

## 3. Risk Factors Implicated in CAPA Development

Importantly, the pathogenesis of IPA differs between neutropenic and non-neutropenic patients, including those with COVID-19, impacting clinical presentation, radiological findings and diagnostic test results in the mycology laboratory [41,42]. Despite these important differences, revised European Organization for Research and Treatment of Cancer/Invasive Fungal Infections Cooperative Group and National Institute of Allergy and Infectious Diseases Mycoses Study Group (EORTC/MSG) definitions [43] focus primarily on neutropenic patients with underlying hematological malignancies and “typical” presentation of IPA and have been shown to have limited applicability and inferior performance in non-neutropenic patients who frequently do not fulfil radiological and host criteria, including patients with COVID-19 [41,44]. This has resulted in the creation of an alternative clinical algorithm for diagnosing IPA in the ICU setting in 2012 [41], which defines putative IPA and is now the standard of care for defining IPA in the ICU [4,45], where highly reliable definitions of IA are still missing (work on improved definitions is currently in progress [45,46]).

Rapid development of CAPA few days following ICU admission [47] resembles the observation made for influenza-associated pulmonary aspergillosis [4,5]. Risk factors predisposing COVID-19 patients to develop secondary pulmonary aspergillosis are similar to those identified for influenza-IPA superinfections [4,5]. The most important risk factors include severe lung damage during the course of COVID-19 [48], the use of corticosteroids in those with ARDS, the widespread use of broad-spectrum antibiotics in intensive care units [49], and the presence of comorbidities such as structural lung defects [47,50,51,52].

There are some reports revealing that pulmonary fibrosis can be triggered by the cytokine storm activated by the viral antigens, toxicity posed by drugs, high airway pressure and hypoxia-induced acute lung injury secondary to mechanical ventilation [53]. While interstitial pulmonary fibrosis per se does not predispose to development of IPA, a small subset of these COVID-19 survivors may require long term corticosteroid treatment, which may predispose them to CAPA years after the acute phase of the viral infection. Overall, 29% of the CAPA cases published to date (10/35) had received systemic corticosteroids (Table 1). In those with ARDS, systemic corticosteroids are used to alleviate the immune responses and prevent cytokine storm [6,54,55,56], but may at the same time increase vulnerability for developing secondary infections [4,5].

Although detailed case series have not reported on antibiotic use among patients, broad-spectrum antibiotics are presumed to be used in 75% of COVID-19 patients admitted to ICU [49]. Since the human gut microbiome is a highly complicated structure of bacteria and fungi, although bacteria are the most diverse constituents, the administration of antibiotics results in perturbation of microbiome steady-state composition, which allows fungi to thrive, and may predispose the host to invasive fungal infections once the immune system becomes impaired [65,66].

Underlying medical conditions may also predispose COVID-19 patients to develop CAPA. Among the 35 CAPA cases published to date (Table 1), hypertension (17/35; 49%), diabetes (9/35; 26%), obesity (8/35; 23%), COPD (5/35; 14%), heart diseases (5/35; 14%), hypercholesterinemia (4/35; 11%), and asthma (3/35; 9%) were among the most prevalent comorbidities observed. While hypertension, coronary heart diseases, and diabetes increase the risk of infection overall [67,68,69], structural lung damage caused by COPD or asthma may particularly predispose patients to develop IPA [70].

## 4. CAPA Prevalence

Several studies from China reported high rates of *Aspergillus* infections among COVID-19 patients. In one study from the Jiangsu province in China, 60/257 COVID-19 (23.3%) patients had throat swab samples that tested positive for *Aspergillus* spp. and were reported as *Aspergillus* co-infections [8]. In another Chinese study from the Zhejiang province 8 of the 104 patients with COVID-19 (7.7%) patients were reported to have IPA although questions remain regarding criteria used for diagnosing IPA in this study (authors state EORTC/MSG criteria were used but all 8 patients seemingly lacked host factors) [71]. Another study from China reported that 27% of the COVID-19 patients (13/48) developed fungal infections but lacked further details [7]. In other reports from China, lower rates of fungal infections were reported ranging between 3.2–5% [54,55,72]. None of those studies have used specific definitions and standardized diagnostic algorithms to identify and define CAPA. In fact, diagnosis of pulmonary aspergillosis is challenging with culture exhibiting limited sensitivity [73,74], and galactomannan testing—the current gold standard—is rarely available in China [75]. As a result, some of these reported rates are likely an underestimate of the real burden of IPA in patients with COVID-19 requiring ICU admission, while other rates may be an overestimation due to potentially misinterpreting *Aspergillus* colonization in the upper respiratory tract as *Aspergillus* infection.

More recently, several studies and case-series from Europe (France, Germany, Belgium, and the Netherlands) have reported high rates of CAPA among COVID-19 cases with ARDS, ranging from 20–35% (Table 1) [47,50,51,52]. The development of CAPA was fairly rapid, with a median of 6 days and range of 3–28 days after ICU admission [47,52]. Moreover, two additional CAPA cases have been reported from Germany [61] and single cases have also been reported from the Netherlands [62], Austria [60], Italy [59], Australia [63], and France [57,58] (Table 1). Among 35 CAPA cases reported to date, there were a total of 5 proven cases [52,59]. The overall mortality rate was 63% (22/35), among whom 4 were female (4/8; 50%) and 14 were male (18/27; 67%). The mortality in case series reported from France, Germany, Belgium and Netherlands ranged between 44.5–66.7% [47,50,51,52]. Of particular importance was the 100% fatality rate of those with underlying diseases reported from the Netherlands, while the two patients without underlying conditions both survived [47]. Noteworthy is the fact that COVID-19 patients presented with ARDS typically fall into the elderly category [6], whereas ARDS in those infected with influenza involves both children <5 years old and elderly >65 years old [76]. The difficulties in diagnosing CAPA, which are outlined in more detail in the next section of this review, may also contribute to increased mortality rates. The most notable example is a study from France [57], where both culture and serology assays were negative for the initial respiratory samples and became only positive after the patient expired [57]. In a case from Italy, initial BALF culture was positive for *A. fumigatus* but the treatment was delayed for two days and only started after the serum galactomannan test became positive [59]. CAPA was later confirmed by autopsy examination [59]. As a result, authors encouraged prompt initiation of systemic antifungal therapy immediately after obtaining positive results even if *Aspergillus* is detected in samples from the upper respiratory tract [59]. Since azole resistance can be associated with a higher mortality rate when compared to patients infected with azole susceptible *A. fumigatus* isolates, it is of paramount importance to use antifungal susceptibility testing to inform targeted antifungal treatment, especially in regions with high azole resistance [77]. Azole-resistant *A. fumigatus* isolates were also persistently recovered from tracheal aspirates during the course of azole treatment in the most recent study from the Netherlands implicated a CAPA case for whom [62]. The azole-resistant *A. fumigatus* isolate (itraconazole, voriconazole, and posaconazole MICs were 16, 2, and 0.5 µg/mL, respectively) harbored a well-known mutation, TR34/L98H [62], presumed to have been acquired from the environment [77]. The in vitro MIC value of the isolate obtained at day 19 (2 mg/L) was higher than the voriconazole serum trough concentration measured on day 17 (1.43 mg/L) and despite switching voriconazole to l-AmB, the patient died due to deteriorating health conditions [62]. Overall, *A. fumigatus* appeared to be the most prevalent *Aspergillus* spp. isolated among respiratory samples with positive culture (26/29; 90%), followed by *A. flavus* (2/29; 7%).

## 5. Diagnostic Workup for Accurate Identification of CAPA

The optimal diagnostic algorithm for diagnosing CAPA is currently unknown, and this question is actively being investigated in an ongoing multinational explorative trial in conjunction with the European Confederation of Medical Mycology (ECMM). The most common methods to date include attempting to recover *Aspergillus spp.* on culture media of bronchoalveolar fluid (BALF) and tracheal aspirate, as well as utilizing serologic biomarker testing such as the conventional Galactomannan (GM) from BALF, tracheal aspirate, and serum specimens. Other diagnostic tests that may prove useful also include *Aspergillus* PCR, serum (1→3)-β-d-glucan (BDG), the *Aspergillus* galactomannan lateral flow assay (LFA) (IMMY, Norman, Oklahoma, USA), and the *Aspergillus*-specific lateral-flow device (LFD) test (OLM Diagnostics, Newcastle Upon Tyne, UK).

In published cases and case series from Germany [50,61], France [51,57,58], Italy [59], Austria [60], Belgium [52], Australia [63], and the Netherlands [47], CAPA was most commonly mycologically diagnosed by either culture from BALF or tracheal aspirate and/or based on a positive GM or LFD from BALF or tracheal aspirate (Table 1). Across published cases, *Aspergillus* culture was positive in 29/35 (83%) of patients; of those with a positive culture and a reported source, 16/29 (55%) were recovered from—often undirected—BALF, 12/29 (41%) from tracheal aspirate, and 1/29 (3%) from sputum. In those where a BALF GM test was performed, 14/23 (61%) had a titer ≥1.5 ODI and 16/23 (70%) a titer ≥0.5 ODI, while 6/28 (21%) of those with serum GM results had a titer > 0.5 ODI. PCR from respiratory specimens or tissue was positive in 10/14 (71%) and LFD from tracheal secretion positive in 1/1 of patients.

Thus, BALF and tracheal aspirate culture and conventional GM testing from BALF appear to be the most promising diagnostic modalities. Still, bronchoscopy can potentially aerosolize virus [78] in patients with COVID-19 infection, thus posing a risk to patients and personnel from SARS-CoV-2 virus. In many centers, the role of bronchoscopy is limited and testing from blood samples may be safer and more optimal and allow also for twice weekly screening which has been implemented in many centers [52], although the low levels of GM positivity from serum in these reports is discouraging, and the sensitivity of serum BDG, which is less specific for IA, was only 44% (4/9).

## 6. CAPA Treatment—Current Paradigm

While it is currently unknown whether antifungal treatment of COVID-19 associated IPA translates into a survival benefit, diagnosis should in most cases trigger early antifungal treatment. Outside the hematologic malignancy setting, voriconazole remains the recommended first-line treatment for IPA [79,80]. However, besides its narrow therapeutic window and the requirement for therapeutic drug monitoring to ensure efficacy and prevent neuro and hepatotoxicity [81], drug–drug interactions may particularly limit the use of voriconazole in the ICU setting [82]. Being metabolized via CYP2C19, CYP2C9, and CYP3A4, voriconazole is among the drugs most frequently associated with major drug–drug interactions in the ICU [83]. Furthermore, it may show interactions with experimental COVID-19 therapies, including hydroxychloroquine, atazanavir, lopinavir/ritonavir and last but not least—although weaker—with remdesivir, which is also a substrate for CYP3A4, although its metabolism is primarily mediated by hydrolase activity [84]. Isavuconazole and liposomal amphotericin B are the primary alternative options for treatment of IPA in the ICU [79]. Compared to voriconazole, isavuconazole shows a more favorable pharmacokinetic profile, and is associated with fewer toxicities. However, it is also metabolized via CYP3A4 and could therefore be problematic, although drug–drug interactions are generally less a problem with isavuconazole than with voriconazole [85,86]. Liposomal amphotericin B is a broadly effective alternative treatment option, however, in the ICU renal insufficiency often complicates initiation or requires discontinuation of this antifungal agent. This concern is particularly relevant for patients infected by SARS-CoV-2 which has shown renal tropism and been described as a frequent cause of kidney injury [87]. While itraconazole is now rarely used to treat invasive aspergillosis, it has been shown to exhibit some antiviral activity, specifically as a cholesterol transport inhibitor, and was effective in a feline coronavirus model [88]. In addition, its novel oral SUBA formulation has great bioavailability [89], and itraconazole may therefore be an alternative option for treating COVID-19 associated IPA, although it shares the problem of drug–drug interactions with other triazoles. While currently available echinocandins are not considered first-line treatment options for invasive aspergillosis due to their limited antifungal activity against *Aspergillus* spp., they are generally well tolerated with limited drug–drug interactions and show at least fungistatic activity against *Aspergillus* hyphae [90]. Furthermore, they synergistic interactions with some other antifungals, making them an excellent choice for combination antifungal therapy [90]. New antifungal classes currently under development, namely fosmanogepix and olorofim [91], may have equal efficacy without the same burden of drug–drug interactions and toxicity, and may therefore overcome the limitations of currently available antifungals and become the preferred treatment options in the near future. If the reported high incidence of COVID-19 associated IPA in ICU patients is confirmed in larger studies, there may be justification for prophylaxis trials, for which not only triazoles and nebulized liposomal amphotericin B [52], but also another novel antifungal currently under development, rezafungin (i.e., once weekly echinocandin with improved activity against *Aspergillus* spp.), may be a candidate [92].

## 7. The Current Challenges and How to Tackle Them

Bacterial, fungal and viral secondary infections or co-infections affect mortality. *Acinetobacter baumanii*, *Klebsiella pneumonia* and *Aspergillus* species are important nosocomial pathogens [93] complicating the disease course. Studies from France [51], Germany [50], Belgium [52], and the Netherlands [47], underline the role of CAPA. Diagnosing co-infections is complex and rapid diagnosis plays a crucial role in this setting [49]. Close monitoring for infection development is needed, as well as longitudinal sampling throughout the disease course using culture dependent and independent techniques. *Aspergillus* antigen and PCR testing of respiratory fluids should be a routine procedure for critically ill patients [94], specifically for those suffering from ARDS [50]. Co-infection with human metapneumovirus has been reported in two of five cases in the German CAPA series [50]. It is unknown whether hospitals caring for COVID-19 test comprehensive respiratory pathogen panels, and to date no analysis of mixed viral infection in COVID-19 patients has been reported. In the context of COVID-19, mixed viral infection may be misinterpreted as presence of innocent bystanders and thus remain underreported. With bronchoalveolar lavage and autopsy regarded as high-risk procedures, key diagnostic instruments are lacking. Autopsy studies are key to understanding pathophysiology of COVID-19 [95] and are critically enlighten interaction between SARS-CoV-2 and different pathogens. With availability of lower respiratory samples, normally obtained by BALF, the quality of microbiological and virological work up would be greatly improved. Inspection of trachea and bronchi is achieved by bronchoscopy, which is critical to find possible *Aspergillus* tracheobronchitis. Thus, physicians face the dilemma of taking the hazard of aerosolization of SARS-CoV-2, risking transmission versus the endeavor of facilitating the optimal diagnosis and treatment to the patients entrusted to their care.

To this day, our understanding of the true impact of *Aspergillus* co-infections remains frustratingly limited. Therefore, guidance on proper management of these high-risk procedures to prevent transmission and super spreading of SARS-CoV-2 is needed. The European Confederation of Medical Mycology initiated national multicenter studies aiming to explore the risk of fungal infections during COVID-19 [94] and is currently working on diagnostic and treatment algorithms. Key goals are to improve the outcome by avoiding misdiagnosis and by initiation of early and targeted antifungal treatment.

## 8. Future Perspectives

We anticipate that autopsies of COVID-19 fatalities will increase and likely prove the clinical relevance of CAPA [96]. Immune dysregulation together with epithelial lung damage stemming from COVID-19 immunopathology is a likely mechanism predisposing for IPA development [97]. IPA will be recognized as important co-infection in patients with severe COVID-19, but incidence will likely vary between different ICU settings. In settings where COVID-19 associated IPA occurs most commonly, screening for IPA in blood and true BALF samples (i.e., obtained via bronchoscopy) will be implemented followed by preemptive treatment in those with mycological evidence of IPA. In other high-incidence settings, clinical antifungal prophylaxis trials will be conducted among COVID-19 patients admitted to the ICU aiming to show a decrease in putative [4] and proven IPA cases, as well as overall mortality. Treatment trials will compare efficacy and safety of new antifungal drugs currently under development with established antifungals, initiating a new era of antifungal treatment.

## Figures and Tables

**Table 1:** Clinical characteristics of COVID-19 patients with pulmonary aspergillosis published before 10 June 2020.

Country (Prevalence) ^COHORT^ [Ref]	Age/Sex	Underlying Conditions	CAPA Classification	Local/Systemic Corticosteroid Use	GM (ODI)/Serum BDG (pg/mL)/qPCR	Species (Voriconazole Susceptibility Pattern)	Treatment ^#^	Outcome
Germany (5/19; 26.3%)^ARDS^ [50]	62/F	Cholecystectomy for cholecystitis, arterial hypertension, obesity with sleep apnea, hypercholesterolemia, ex-smoker, COPD (GOLD 2)	Putative	Inhaled steroids for COPD	GM Serum negative GM BALF> 2.5 qPCR BALF = Positive	*Aspergillus fumigatus* (S) culture from BALF	VCZ	Died
	70/M	Vertebral disc prolapse left L4/5, flavectomy and nucleotomy, Ex-smoker	Putative	No	GM Serum = 0.7 GM BALF> 2.5 qPCR BALF = Positive	*A. fumigatus* by PCR; negative culture	ISA	Died
	54/M	Arterial hypertension, diabetes mellitus, aneurysm coiling right *A. vertebralis*	Putative	Intravenous corticosteroid therapy 0.4 mg/kg/d, total of 13 days)	GM Serum negative GM BALF> 2.5 qPCR BALF = Positive	*A. fumigatus* (S) culture from tracheal aspirate	CASPO→ VCZ	Alive
	73/M	Arterial hypertension, bullous emphysema, smoker, COPD (GOLD 3), Previous Hepatitis B	Putative	Inhaled steroids for COPD	GM Serum negative qPCR tracheal secretion = Positive	*A. fumigatus* (S) culture from tracheal aspirate	VCZ	Died
	54/F	None	Putative	No	GM Serum = 1.3 and 2.7 qPCR tracheal secretion = Negative	Negative culture	CASPO→ VCZ	Alive
France (9/27; 33.3%)^ARDS^ * [51]	53/M	Hypertension, obesity, ischemic heart disease	Putative	Dexamethasone iv 20 mg once daily from day 1 to day 5, followed by 10 mg once daily from day 6 to day 10	GM Serum = 0.13 GM BALF = 0.89 BDG = 523 qPCR = Negative	Negative culture	None	Alive
	59/F	Hypertension, obesity, diabetes	Putative	No	GM Serum = 0.04 GM BALF = 0.03 qPCR = Negative	*A. fumigatus*, culture from BALF	None	Alive
	69/F	Hypertension, obesity	Putative	Dexamethasone iv 20 mg once daily from day 1 to day 5, followed by 10 mg once daily from day 6 to day 10	GM Serum = 0.04 BDG = 7.8 qPCR BALF = 23.9	*A. fumigatus*, culture from tracheal secretion	None	Alive
	63/F	Hypertension, diabetes, ischemic heart disease	Putative	Dexamethasone iv 20 mg once daily from day 1 to day 5, followed by 10 mg once daily from day 6 to day 10	GM Serum = 0.51 GM BALF = 0.15 BDG = 63	Negative culture	None	Died
	43/M	Asthma with steroid use history	Putative	No	GM Serum = 0.04 GM BALF = 0.12 BDG = 7 qPCR = Negative	*A. fumigatus*, culture from BALF	None	Alive
	79/M	Hypertension	Putative	Dexamethasone iv 20 mg once daily from day 1 to day 5, followed by 10 mg once daily from day 6 to day 10	GM Serum = 0.02 GM BALF = 0.05 BDG = 23 qPCR BALF = 34.5	*A. fumigatus*, culture from BALF	None	Alive
	77/M	Hypertension, asthma	Putative	Dexamethasone iv 20 mg once daily from day 1 to day 5, followed by 10 mg once daily from day 6 to day 10	GM Serum = 0.37 GM BALF = 3.91 BDG = 135 qPCR BALF = 29	*A. fumigatus*, culture from BALF	VCZ	Died
	75/F	Hypertension, diabetes	Putative	Dexamethasone iv 20 mg once daily from day 1 to day 5, followed by 10 mg once daily from day 6 to day 10	GM Serum = 0.37 GM BALF = 0.36 BDG = 450 qPCR BALF = 31.7	*A. fumigatus*, culture from BALF	CASPO	Died
	47/M	Multiple myeloma with steroid therapy	Probable	No	GM Serum = 0.09 BDG = 14	*A. fumigatus*, culture from tracheal secretion	None	Died
Netherlands (6/31; 19.4%)^ARDS^ [47]	83/M	Cardiomyopathy	Possible	Prednisolone 0ꞏ13 mg/kg/day for 28 days pre-admission	GM Serum = 0.4	*A. fumigatus*, culture from tracheal aspirate	VCZ + ANID (5/6) L-AmB (1/6)	Died
	67/M	COPD (GOLD 3), Post RTx NSCLC 2014	Possible	Prednisolone 0ꞏ37 mg/kg/day for 2 days pre-admission	NA	*A. fumigatus*, culture from tracheal aspirate		Died
	75/M	COPD (GOLD 2a)	Probable	No	GM BALF = 4.0	*A. fumigatus*, culture from BALF		Died
	43/M	None	Probable	No	GM Serum = 0.1 GM BALF = 3.8	NA		Alive
	57/M	Bronchial asthma	Probable	Fluticasone 1ꞏ94 mcg/kg/day for 1 month pre-admission	GM Serum = 0.1 GM BALF = 1.6	*A. fumigatus*. culture from BALF		Died
	58/M	None	Possible	No	NA	*Aspergillus spp.* (S), culture from sputum		Alive
Belgium (7/20; 35%)^ARDS^ [52]	86/M	Hypercholesterinemia	NA	No	GM serum = 0.1	*A. flavus* culture from tracheal aspirate	None	Died
	38/M	Obesity, hypercholesterinemia	Proven	No	GM serum = 0.3 GM BALF > 2.8	*A. fumigatus* culture from BALF	VCZ, ISA	Alive
	62/M	Diabetes	Proven	No	GM serum = 0.2 GM BALF = 2	*A. fumigatus* culture from BALF	VCZ	Died
	73/M	Diabetes, obesity, hypertension, hypercholesterinemia	Proven	No	GM serum= 0.1 GM BALF > 2.8	*A. fumigatus* culture from BALF	VCZ	Alive
	77/M	Diabetes, chronic kidney disease, hypertension, pemphigus foliaceus	Proven	Yes, ND	GM serum = 0.1 GM BALF = 2.79	*A. fumigatus* culture from BALF	VCZ	Alive
	55/M	HIV, hypertension, hypercholesterinemia	NA	No	GM serum = 0.80 GM BALF = 0.69	Negative culture	VCZ, ISA	Died
	75/M	Acute myeloid leukemia	NA	No	GM BALF = 2.63	*A. fumigatus* culture from BALF	VCZ	Died
France (1)^ARDS^ [57]	74/M	Myelodysplastic syndrome, CD8 ^+^ T-cell lymphocytosis, Hashimoto’s thyroiditis, hypertension, benign prostatic hypertrophy	Putative	No	First GM on tracheal secretion = Negative First qPCR = Positive Second GM tracheal secretion = NA Second qPCR = Positive Direct smear of the second sample = branched septate hyphae	*A. fumigatus*, culture of the second tracheal secretion	None	Died
France (1/5; 20%)^Mixed ICU^ [58]	80/M	Thyroid cancer (patient presented with ARDS)	Putative	NA	No	*A. flavus*, culture from tracheal secretion	VCZ→ ISA	Died
Italy (1)^ARDS^ [59]	73/M	Diabetes, hypertension, obesity, hyperthyroidism, atrial fibrillation	Proven	No	GM Serum = 8.6 qPCR from paraffin block tissue = Positive	*A. fumigatus*, culture from BALF	L-AmB → ISA	Died
Austria (1)^ARDS^ [60]	70/M	COPD (GOLD 2), obstructive sleep apnea syndrome, insulin-dependent type 2 diabetes with end organ damage, arterial hypertension, coronary heart disease, and obesity	Putative	Inhaled Budesonide (400 mg per day)	GM Serum = Negative BDG = Negative LFD Positive from endotracheal aspiration	*A. fumigatus*, culture from endotracheal aspiration	VCZ	Died
Germany (2)^ARDS^ [61]	80/M	Suspected pulmonary fibrosis	ND	No	GM Serum = 1.5 GM BALF = 6.3	*A. fumigatus*, culture from BALF	L-AmB	Died
	70/M	None	ND	No	GM Serum = Negative GM BALF = 6.1	*A. fumigatus*, culture from BALF	L-AmB	Died
Netherlands (1)^ARDS^ [62]	74/F	Polyarthritis, reflux, stopped smoking 20 years ago	Putative	No	GM serum = Persistently < 0.5 GM tracheal aspirate = >3 BDG serum = 1590	*A. fumigatus*, culture from tracheal aspirate (R)^TR34/L98H^ICZ = 16µg/mL, VCZ = 2µg/mL, and POSA = 0.5µg/ml	VCZ + CASPO→ Oral VCZ→ L-AmB	Died
Australia (1) ^ARDS^ [63]	66/F	Hypertension, osteopenia, ex-smoker (20 pack years)	Putative	No	N/A	*A. fumigatus* culture from tracheal aspirate (3x)	VCZ + Therapeutic Drug monitoring	Alive

* All serum qPCR remained negative. # All dosages are standard dosages (e.g., VCZ 6 mg/kg bid Day 1, and 4 mg/kg bid starting Day 2) [64]. ARDS: acute respiratory distress syndrome; NA: not applicable; ND: not determined; BALF: bronchoalveolar lavage fluid; BDG: beta-D-Glucan; COPD: chronic obstructive pulmonary disease; GM; galactomannan; GOLD: global initiative for chronic obstructive lung disease; NSCLC: non-small-cell lung cancer; ODI: optical density index; RTx: radio therapy; LFD: lateral flow device; qPCR: quantitative real-time PCR; VCZ: voriconazole; ISA: isavuconazole; CASPO: caspofungin; ANID: anidulafungin; L-AmB: liposomal amphotericin B; ICZ: itraconazole; POSA = posaconazole.
